# The utility of mouse models to provide information regarding the pathomolecular mechanisms in human genetic skeletal diseases: The emerging role of endoplasmic reticulum stress (Review)

**DOI:** 10.3892/ijmm.2015.2158

**Published:** 2015-03-30

**Authors:** MICHAEL D. BRIGGS, PETER A. BELL, KATARZYNA A. PIROG

**Affiliations:** Institute of Genetic Medicine, Newcastle University, International Centre for Life, Newcastle upon Tyne, NE1 3BZ, UK

**Keywords:** skeletal dysplasia, cartilage, endoplasmic reticulum stress, mouse models, disease mechanisms, pseudoachondroplasia, multiple epiphyseal dysplasia

## Abstract

Genetic skeletal diseases (GSDs) are an extremely diverse and complex group of rare genetic diseases that primarily affect the development and homeostasis of the osseous skeleton. There are more than 450 unique and well-characterised phenotypes that range in severity from relatively mild to severe and lethal forms. Although individually rare, as a group of related genetic diseases, GSDs have an overall prevalence of at least 1 per 4,000 children. Qualitative defects in cartilage structural proteins result in a broad spectrum of both recessive and dominant GSDs. This review focused on a disease spectrum resulting from mutations in the non-collagenous glycoproteins, cartilage oligomeric matrix protein (COMP) and matrilin-3, which together cause a continuum of phenotypes that are amongst the most common autosomal dominant GSDs. Pseudoachondroplasia (PSACH) and autosomal dominant multiple epiphyseal dysplasia (MED) comprise a disease spectrum characterised by varying degrees of disproportionate short stature, joint pain and stiffness and early-onset osteoarthritis. Over the past decade, the generation and deep phenotyping of a range of genetic mouse models of the PSACH and MED disease spectrum has allowed the disease mechanisms to be characterised in detail. Moreover, the generation of novel phenocopies to model specific disease mechanisms has confirmed the importance of endoplasmic reticulum (ER) stress and reduced chondrocyte proliferation as key modulators of growth plate dysplasia and reduced bone growth. Finally, new insight into related musculoskeletal complications (such as myopathy and tendinopathy) has also been gained through the in-depth analysis of targeted mouse models of the PSACH-MED disease spectrum.

## 1. Skeletal dysplasias resulting from mutations in cartilage structural proteins

Qualitative (i.e., dominant-negative or antimorphic mutations) defects in cartilage structural proteins result in a diverse range of both recessive and dominant genetic skeletal dysplasias (Table I). These structural proteins include cartilage collagens (types II, IX and XI), proteoglycans (aggrecan) and glycoproteins [cartilage oligomeric matrix protein (COMP) and matrilin-3 (MATN3)] and many of the mutations are predicted to affect the folding and/or function of these important molecules. This review specifically concentrates on those diseases resulting from mutations in the non-collagenous glycoproteins, COMP and matrilin-3, which together cause a continuum of phenotypes that are amongst the most common autosomal dominant skeletal dysplasias.

*The pseudoachondroplasia (PSACH) and multiple epiphyseal dysplasia (MED) disease spectrum: the generation and study of genetic mouse models*. Originally believed to be different but clinically related phenotypes, it was gene and mutation identification in the 1990s that demonstrated that PSACH and some forms of MED were allelic diseases resulting from mutations in *COMP* (1). However, MED is genetically heterogeneous and autosomal dominant forms can also result from mutations in the genes encoding matrilin-3 and type IX collagen (2) (Table I).

Despite the first mutations being identified in *COMP*, *MATN3* and the type IX collagen genes in the six years between 1995 and 2001 (Table I), very little progress has been made in understanding disease mechanisms despite numerous cell culture studies (reviewed in ref. 3). A lack of relevant pathological tissues (such as cartilage growth plate), and the obvious limitations of cell culture models to study long bone growth, meant that only small incremental increases in knowledge were possible.

Over the past eight years, however, a number of different genetic approaches have been used to generate mouse models of PSACH-MED that recapitulate the various phenotypes and allow disease mechanisms to be studied in detail *in vivo* (Table II). Moreover, the generation of novel phenocopies to model specific disease mechanisms have confirmed the importance of endoplasmic reticulum (ER) stress and chondrocyte proliferation as key modulators of growth plate dysplasia. Finally, new insight into related musculoskeletal complications (such as myopathy and tendinopathy), which may be of clinical utility, has also been gained through the in-depth analysis of targeted mouse models of the PSACH-MED disease spectrum.

## 2. Knock-in mouse models provide new insight into disease mechanisms

The application of homologous recombination and *Cre*-lox technology has allowed the generation of a series of mouse lines that genetically modelled phenotypes within the PSACH-MED disease spectrum, thereby allowing key pathological findings to be described and disease mechanisms to be studied in detail (Tables II and III).

In the first instance, an allelic series of PSACH models were generated representing the two major classes of *COMP* mutations: the type III repeat (T3) region and the carboxyl-terminal domain (CTD) (2,4). Type III repeat region mutations account for approximately 85% of all COMP-related PSACH-MED, whereas CTD mutations represent 15% of cases (2). The mutations that were selected to represent these two different classes were the common in-frame deletion of an aspartic acid residue from the C-type motif of repeat T3_7_ (p.Asp469del), which accounts for 30% of all PSACH, and the recurrent p.Thr585Met missense mutation in the CTD (2,4). The p.Thr585Met mutation has been reported in four different studies and results in a phenotypic spectrum ranging from MED to mild PSACH (4). Previous studies using cell models consistently demonstrated the retention of mutant Asp469del COMP in the ER (3). By contrast, p.Thr585Met, along with several other CTD mutations, was efficiently secreted into the culture media of various cell models (5,6). These early cell model studies have suggested different disease mechanisms between these two classes of mutations that justified validation and in-depth analysis using relevant mouse models.

### The p.Thr585Met (Comp^T585M^) model of mild PSACH/severe MED

Individuals with p.Thr585Met [and several other CTD mutations (4)] often present with a mild form of PSACH and this was reflected in the *Comp*^T585M^ mouse model which showed only a 5% reduction in bone length at 9 weeks of age (7). This is consistent with previous family studies, which demonstrated that individuals with the Thr585Met mutation may have average stature, despite having significant epiphyseal and metaphyseal changes in the large joints (8).

**Figure 1:**
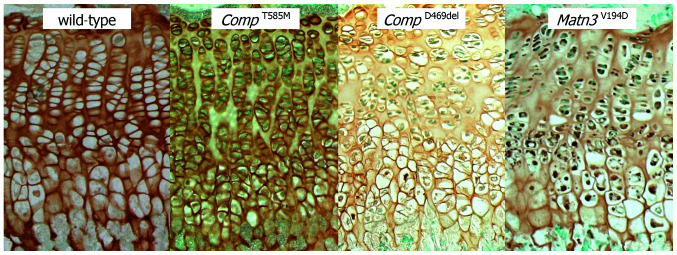
Organization of the growth plate in mutant mice is disrupted by 3 weeks of age and shows marked hypocellularity along with the retention and/or mislocalisation of cartilage structural proteins. Representative immunohistochemistry (IHC) of the growth plates from 3 week-old wild-type and mutant mice showing disruption to chondrocyte columns in mice homozygous for the *Comp* p.Thr585Met (*Comp*^T585M^), *Comp* p.D469del (*Comp*^D469del^) and *Matn3* Val194Asp (*Matn3*^V194D^) mutations. IHC using COMP (wild-type, *Comp*^T585M^ and *Comp*^D469del^) and matrilin-3 (*Matn3*^V194D^) antibodies revealed less staining in the extracellular matrix (ECM) between the proliferating columns in the growth plates of mice carrying all three mutations. Furthermore, there was intracellular staining for mutant COMP and matrilin-3 in chondrocytes from the *Comp*^D469del^ and *Matn3*^V194D^ mice, respectively.

**Figure 2:**
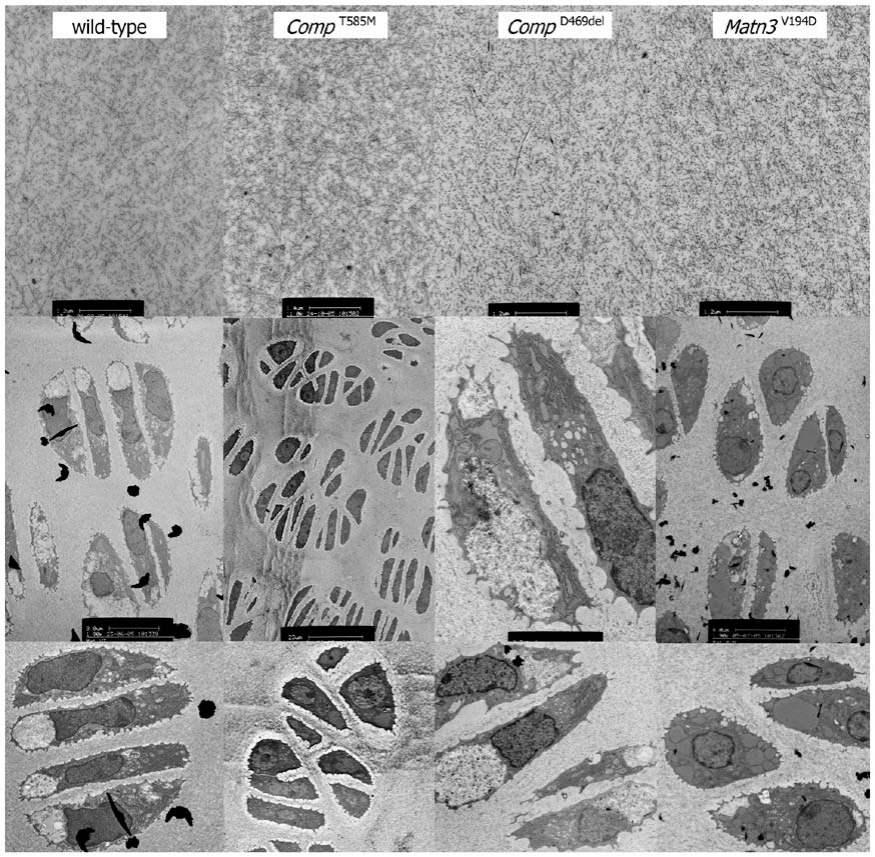
Chondrocytes have dilated cisternae of endoplasmic reticulum (ER) and the extracellular matrix (ECM) ultrastructure is altered in mutant growth plates. These representative images were taken from a complete TEM montage of a day 7 tibia growth plate (from resting to mineralisation zones) and show chondrons from the proliferative zone of mice homozygous for the *Comp* p.Thr585Met (*Comp*^T585M^), *Comp* p.D469del (*Comp*^D469del^) and *Matn3* Val194Asp (*Matn3*^V194D^) mutations. Top panel, the ultrastructure of the interterritorial matrix at 1 week of age is altered in all three mutant growth plates and is characterised by more prominent appearing collagen fibrillar material. Scale bars, 1.2 *μ*m (wild-type, *Comp*^D469del^ and *Matn3*^V194D^) or 1.4 *μ*m (*Comp*^T585M^). Middle and bottom panels, chondrocytes from *Comp*^D469del^ and *Matn3*^V194D^ mice which show enlarged individual cisternae of ER compared to the wild-type mice in which the ER displays has the typical ribbon appearance. The *Comp*^T585M^ growth plate shows misaligned and abnormally shaped chondrocytes despite mutant T585M COMP being secreted.

Immunohistochemistry and electron microscopy confirmed a lack of mutant protein retention in the ER (Figs. 1 and 2), which is consistent with previous cell culture studies (5,6). However, there were severe disruptions to the morphology of the growth plate and to the size, shape and arrangement of individual chondrocytes and chondrons (7). The composition of the growth plate extracellular matrix (ECM) was also disrupted with changes to the localisation of several ECM proteins and the under-sulfation of proteoglycans (7). Proteomic interrogation confirmed changes to the extractability of several ECM proteins, including collagens [α1(XIV), α1(IX), α1(XI), α1(XII) and α6(VI)], laminin β2 and fibronectin (9). Finally, there was a significant reduction in chondrocyte proliferation that was accompanied by increased and spatially dysregulated apoptosis (7) (Table III).

### The p.Asp469del (Comp^D469del^) model of typical severe PSACH

p.Asp469del is the most common *COMP* mutation and invariably results in typically severe PSACH (4,5). Numerous cell culture studies have been undertaken on this archetypal mutation, which have consistently shown that p.Asp469del results in the retention of mutant COMP protein within the rough ER (rER) of cells, along with matrilin-3 and collagen type IX (10,11). Furthermore, cartilage samples from PSACH patients appear to have a disorganised ECM and there is an increase in cell death *in vivo* (12) that can be recapitulated *in vitro* by several different cell culture models (13,14).

*Comp*^D469del^ mice were normal at birth, but grew slower than their wild-type littermates and developed a progressive short-limb dwarfism, which was characterised by a 5–6% reduction in final tibia and femur lengths. Moreover, the mutant mice developed a hip dysplasia that was characterised by an increase in the angle of deflection from the vertical of the tuberosity of the ischium (15). The reductions in the final bone lengths of *Comp*^D469del^ mice were less than would be expected for a phenotypically exact model of typical severe PSACH and may be best explained by the mixed genetic background of the mutant mouse strain.

Notwithstanding the relatively mild reduction in final bone lengths of *Comp*^D469del^ mice, there was significant growth plate pathology that was illustrative of the hallmarks of PSACH (15) (Table III). In particular, chondrocyte columns were reduced in number and poorly organised in the growth plates of *Comp*^D469del^ mice, whilst mutant COMP was retained within the ER of chondrocytes at all stages of differentiation (15) (Fig. 1). The composition and appearance of the ECM was disrupted, with changes to the localisation of several ECM proteins, including the co-retention of matrilin-3 and type IX collagen (15). Proteomic interrogation confirmed changes to the extractability of several ECM proteins, including throm-bospondin-3 and -4, several collagens [α1(XIV), α1(IX) and α1(VI)], tenascin-C and -X and epiphycan (9). Most noticeably, chondrocyte proliferation was significantly reduced and apoptosis was greatly increased in the proliferative zone and spatially dysregulated throughout the entire growth plate (15) (Table III).

### The p.Val194Asp (Matn3^V194D^) model of moderate MED

Missense mutations and small in-frame deletions in the von Willebrand Factor A (vWFA) domain of matrilin-3 cause moderate to severe forms of MED (2). The various missense mutations are distributed between the β-sheet (~85%) and α-helical (~15%) regions of the vWFA domain. Mutations in the β-sheet regions primarily disrupt the folding of the vWFA domain, resulting in the retention of mutant protein in the ER of cells (16). By contrast, some α-helical mutations allow the correct folding and secretion of mutant matrilin-3 (17); however, there are exceptions to these generalisations. The *MATN3* mutation (p.Val.194Asp) selected for mouse model generation was the first EDM5 mutation to be identified and is an archetypal example (18).

*Matn3*^V194D^ mice were normal at birth but developed short-limbed dwarfism that was characterised by a final reduction in tibia length of 12–13% (19). There was a significant retention of mutant matrilin-3 in the growth plate chondrocytes, which were abnormal in shape and resulted in poorly organised chondrons and chondrocyte columns (Figs. 1 and 2). Chondrocyte proliferation was significantly reduced and whilst there was no overall increase in apoptosis it was noticeably dysregulated with increased cell death occurring throughout the entire hypertrophic zone (19) (Table III). Changes to the appearance of the cartilage ECM may be partly attributable to differences in the extractability of various collagens [α1(XIV) and α3(VI)], osteomodulin, biglycan, tenascin-C and epiphycan (9).

## 3. Transgenic approaches for generating mouse models

Several transgenic approaches have also been used to generate mouse models of the common PSACH mutation, which do not rely on the targeted integration of a single copy of the mutant allele (Table II).

### Standard transgenic approaches

In the first example, a transgenic mouse model was generated by Schmitz *et al*, in which rat Asp469del COMP cDNA expression was achieved through the *Col2a1* promoter (20). Mutant COMP was over-expressed by ~40% and transgenic mutant male mice were up to 8% shorter than transgenic wild-type mice at 6 months of age. In an attempt to exacerbate this relatively mild phenotype, the transgenic mutant mice were crossed onto a *Comp*-null line to ensure that only rat COMP Asp469del was expressed by chondrocytes. This cross resulted in a male-specific increase in disease severity that was characterised by a further decrease in body and femur lengths. Chondrocytes within the proliferative zone showed the retention of the transgenic mutant COMP, the levels of which were further increased on the *Comp*-null background (20). Moreover, there were abnormal changes to the morphology of the cartilage ECM, including the loss of proteoglycans and changes to the localisation of other ECM molecules, such as type VI collagen, aggrecan and matrilin-1. Finally, increased apoptosis was observed in combination with distinct areas of hypocellularity and these key pathological features are now recognised as hallmark descriptors of the PSACH growth plate *in vivo* (20).

Subsequently, Posey *et al* hypothesised that the overexpression of Asp469del COMP would best recapitulate the PSACH disease phenotype in a mouse model and therefore used the inducible overexpression of mutant *Comp* that was driven from the *Col2a1* promoter (21). One reason for taking this approach was as a consequence of limited phenotypic and pathological effects observed in earlier PSACH transgenic mice models (3), which highlights the unpredictability of standard transgenic approaches for developing mouse models of human genetic skeletal diseases (GSDs) (Table II).

In the first model, a BAC clone containing human *COMP* and its native promoter was used to generate two founders (1 male and 1 female) of which only the female demonstrated a clinical phenotype and both founders died prematurely (3). In a second approach, Asp469del COMP expression from human cDNA was driven by a mouse *Col2a1* promoter and enhancer. Genomic analysis demonstrated that 12–20 copies of the transgene had integrated into mouse chromosome 10. Tibia measurements confirmed a 6% reduction in final length and there was some disorganization of the growth plate, with fewer chondrocytes organised into columns of chondrons; however, in contrast to the study by Schmitz *et al* (20), no intracellular retention of mutant Asp469del COMP was observed in growth plate chondrocytes (3).

### Bigenic inducible overexpression of mutant Comp Asp469del

The most recent transgenic approach involved the generation of bigenic mice in which the expression of *Comp* Asp469del was controlled by a tetracycline responsive element promoter, which was itself under the control of the tetracycline trans-activator coding sequence with a *Col2a1* promoter to ensure cartilage-specific expression (21). Whilst *Col2a1*-driven overexpression of *Comp* Asp469del was assumed to be robust, the levels of mutant *Comp* mRNA were only determined at E15 and shown to be 4- to 6-fold higher than endogenous *Comp* mRNA (21). Unfortunately, the relative levels of *Comp* Asp469del mRNA and protein were not determined at later time-points, thus limiting the interpretation of phenotypic and pathological readouts, particularly when these analyses were performed postnatally. Nevertheless, key pathological features such as mutant COMP retention, growth plate dysplasia and marginally increased chondrocyte apoptosis were identified in this mouse model (21). Subsequent developmental analysis of this mouse model (22) confirmed growth plate dysplasia by 3 weeks of age that was characterised by disorganization, reduced thickness and distinct areas of hypocellularity. Mutant Asp469del COMP retention was shown to be progressive until 2 weeks of age. Analysis of chondrocyte death by terminal deoxynucleotidyltransferase-mediated dUTP nick-end labelling (TUNEL) confirmed that abnormally increased apoptosis was occurring in the hypertrophic zone by 2 weeks of age, and increased further with age (~3–4-fold); however, the precise localisation of increased apoptosis within the different growth plate zones was not determined. Long bone growth was significantly reduced by 12% in the tibia; however, skull and snout lengths were also shown to be smaller than wild-type controls, indicating that intramembraneous ossification was also disrupted by the transgenic overexpression of mutant COMP, which is not consistent with other mouse models or individuals with PSACH. Moreover, the pathophysiological relevance of *Comp* Asp469del overexpression is not known and whilst strong phenotypic effects were observed, the genetic pathways that are induced by this transgenic approach may not necessarily be representative of the human disease.

## 4. Activation of canonical and/or novel ER stress pathways is genotype-specific

The expression of *Comp*^D469del^ and *Matn3*^V194D^ results in ER retention of the relevant mutant proteins and the co-retention of interacting partners that ultimately results in ER stress (15,19); however, the downstream stress pathways that are activated are genotype-specific.

For example, the expression of *Matn3*^V194D^ induces the activation of the canonical unfolded protein response (UPR), which is characterised by the upregulation of binding immunoglobulin protein (BiP) (a sentinel marker of UPR) and a broad range of chaperones, foldases and protein disulphide isomerases (23). Moreover, the downstream splicing of X-box binding protein 1 (Xbp1) in *Matn3*^V194D^ chondrocytes (and also in cell models) is symbolic of classical UPR; however, this prolonged ER stress does not result in a C/EBP-homologous protein (CHOP)-mediated increase in apoptosis (23) (and our unpublished observations).

In contrast to mutant matrilin-3, the ER retention of *Comp*^D469del^ does not induce a canonical UPR, but a novel form of ER stress that is characterised by changes in the expression of groups of genes implicated in oxidative stress, cell cycle regulation and apoptosis (15). Similar UPR-independent pathways, such as the ER overload response (EOR), appear to be associated with the aggregation of mutant proteins in the ER that eventually induce toxic gain-of-function (24). This has been noted with serpinopathies in which the aggregation of insoluble misfolded α1-antitrypsin triggers an alternative ER stress response that is independent of the UPR and involves the activation of nuclear factor-κB (NF-κB) signalling (25). Moreover, it has been proposed that an aggregating protein response (APR) is activated by glycine substitutions in the triple helical regions of type I collagen that also do not induce canonical UPR (26,27).

Our own studies have identified differences in the molecular organization of the mutant COMP and matrilin-3 proteins that are retained in the ER of mouse chondrocytes and cell culture models (9,28). Mutant matrilin-3 forms non-native disulphide bonded aggregates, due to a delay in the folding of the single vWFA domain, which may render it inaccessible to degradation. By contrast, mutant D469del COMP does not appear to aggregate and is retained in the ER in its apparent native state of either tetramers or pentamers (9). This difference in the molecular organization of mutant protein aggregates may play a role in determining which ER stress pathways are activated.

These unexpected findings demonstrate that ER stress, as a result of mutant protein misfolding, and the genetic pathways that are activated in an attempt to restore ER homeostasis, are likely to be more diverse in mouse models and human genetic diseases than in the more commonly studied chemically-induced ER stress experimental systems (29). However, despite the downstream activation of several potential pathways, such as UPR, APR and/or EOR, a common consequence appears to be the reduction in chondrocyte proliferation (Table III).

## 5. Common genetic pathways identified in the different mouse models of Asp469del COMP pseudoachondroplasia

Despite differences in the relative severity of growth plate dysplasia, chondrocyte cell death and long bone growth between the different *Comp* Asp469del mouse models (Table II), transcriptomic profiling has nevertheless implicated several genetic pathways in common (15,22).

The microarray analysis of mRNAs isolated from chondrocytes of the *Comp*^D469del^ knock-in mouse model showed a complex disease profile with expression changes in groups of genes implicated in oxidative stress, cell cycle regulation and apoptosis, which is consistent with the chondrocyte and growth plate pathology (15). Moreover, this study demonstrated that reduced cell proliferation and increased dysregulated chondrocyte apoptosis was associated with the downregulation of peroxiredoxin 2, which is a gene important for protecting cells against oxidative damage and also in regulating apoptosis (15). Similarly, transcriptomic analysis of mRNAs isolated from the chondrocytes of the bigenic transgenic mouse identified cell cycle regulation, inflammation, oxidative stress and DNA damage as the major classes of genes implicated in disease pathogenesis (22).

## 6. Missense mutations in matrilin-3 and COMP cause changes in the extractability of other cartilage proteins and influence ECM organization

Analysis of the cartilage proteome of *Matn3*^V194D^, *Comp*^D469del^ and *Comp*^T585M^ mice has identified differences in the extractability of numerous structural ECM components relative to the controls (9). The extraction of type IX collagen [a fibril-associated collagen with interrupted triple helices (FACIT)] was increased in *Comp*^D469del^ and *Comp*^T585M^ mice, which may alter the integrity of the II/IX/XI heterofibrillar collagen network. Other changes in the extractability of FACIT collagens were observed in *Matn3*^V194D^ (i.e., the decreased extraction of collagen types XII and XIV), *Comp*^D469del^ (decrease in types IX and XIV) and *Comp*^T585M^ (decrease in types IX and XII, but increase in type XIV) cartilage, which further implied disruption to ECM organization as a consequence of *Comp* and *Matn3* mutations that correlated with the observations of altered ECM morphology detected by electron microscopy (7,15,19) (Fig. 2).

In addition to these commonalities between mutant mouse models, discrete patterns of ECM protein extraction were also observed (9). Most notably, the extraction of tenascin X was greatly reduced from *Comp*^D469del^ cartilage; however, the functional significance of this, and also the increases in tenascin C extractability observed in *Comp*^D469del^ and *Matn3*^V194D^ mice, remains to be determined.

## 7. Novel phenocopies of chondrocyte-specific ER stress provide information regarding intracellular disease mechanisms and the influence of chondrocyte proliferation

Defining the relative contributions of intracellular disease mechanisms (ER stress) and extracellular defects (ECM disruption) to the initiation and progression of growth plate dysplasia is experimentally challenging. Therefore, to directly test the role of ER stress in growth plate dysplasia and reduced long bone growth, independent of disruptions to the cartilage ECM, a novel transgenic approach was taken. This approach was previously established and validated as an innovative tool to dissect disease mechanisms in metaphyseal chondrodysplasia type Schmid, which can be caused by missense mutations in the gene encoding type X collagen (30).

To delineate the relative influence of ER stress to the development of chondrodysplasia, the expression of a G2320R mutant form of thyroglobulin (*Rdw*) was targeted primarily to resting and proliferating chondrocytes using the *Col2a1* promoter (31). The expression and retention of this mutant exogenous protein in the rER of growth plate chondrocytes resulted in chronic cell stress and reduced bone growth, but without inducing any disruptions to the architecture and overall organization of the ECM. More significantly, however, decreased bone growth appeared to be the direct result of reduced chondrocyte proliferation in the growth plates in transgenic mice.

## 8. Reduced chondrocyte proliferation is a shared cellular response to all forms of ER stress and defines a common denominator of PSACH-MED pathology

The pivotal role of ER stress in the initiation of growth plate dysplasia and abnormal bone growth was revealed through targeted knock-in mouse models and novel transgenic phenocopies (Table II). Whilst it has been previously suggested that increased and dysregulated chondrocyte apoptosis is the major cause of reduced bone growth in PSACH (22), our recent analysis of the *Tg*^Rdw^ transgenic phenocopy has demonstrated that reduced chondrocyte proliferation alone is sufficient to cause a significant reduction in bone length in the absence of increased apoptosis or extensive disruption to cartilage ECM composition and assembly (31). We therefore hypothesise that modulating chondrocyte proliferation is likely to exert the greatest influence on long bone growth in PSACH-MED and this may represent an attractive target for therapy. Indeed, dysregulated cyclin D1 signaling during the G1 phase of the cell-cycle causes reduced chondrocyte proliferation in a mouse model of diastrophic dysplasia due to a p.A386V substitution in the eighth transmembrane domain of solute carrier family 26 member 2 (*Slc26a2*) (32). Moreover, recent mouse model studies have demonstrated that long bone length can be restored in achondroplasia by targeting the mitogen-activated protein kinase (MAPK) pathway, which is activated by the tyrosine kinase receptor *FGFR3* mutations in achondroplasia, and causes a disruption to normal chondrocyte differentiation (33). Finally, transgenic expression of mutant forms of type II collagen causes ER-stress induced chondrodysplasia (34,35), which can also defined by reduced chondrocyte proliferation and may be associated with disrupted cell polarity and the abnormal organization and morphology of the primary cilia (35).

## 9. Genetic background influences phenotypic severity in the *Comp*^T585M^ mouse model of PSACH-MED by impacting on chondrocyte proliferation and apoptosis

Several clinical genetic studies have demonstrated that there is considerable intra- and interfamilial variability in disease presentation in both PSACH and MED, which strongly points to the existence of genetic modifiers of phenotypic severity. This is best highlighted by several *MATN3* mutations, such as Arg121Trp, which are associated with marked interfamilial variability in the radiographic phenotype of patients (36). Patients carrying the T585M COMP mutation also show variability in final adult height (8).

In-bred mouse strains are a useful tool which can be used to study complex disease traits when the influence of a genetic modifier is suspected (37). Interestingly, crossing the *Comp*^T585M^ knock-in mice (originally on a mixed genetic background) onto a C57BL6/J inbred background has been shown to increase the severity of the phenotype by further disrupting chondrocyte proliferation, apoptosis and matrix deposition (38). Moreover, mutant COMP was retained inside the rER of chondrocytes, which was a feature not observed in the original mouse model (7). Dixon and Dixon (37) demonstrated a similar phenotypic variability in their model of craniofacial abnormalities in Treacher Collins Syndrome that is genetic background-dependent. This feature renders inbred mouse strains a valuable genetic model for quantitative trait loci (QTL) studies and the analysis of genetic modifiers co-segregating with various disease traits.

## 10. Insight into additional musculoskeletal complications of PSACH-MED revealed for the first time through the in-depth analysis of mouse models

Several skeletal dysplasias, including PSACH and MED, often result from mutations in the extracellular proteins that are synthesised by cells of the mesenchymal lineage (39). It is therefore not surprising that patients may present with additional related musculoskeletal complications (40). Mild PSACH/MED patients are sometimes diagnosed with mild myopathy or a ‘neuromuscular problem of unknown etiology’ prior to the confirmation of a chondrodysplasia. Indeed, we have previously reported several patients who initially presented with excessive fatigue during walking; difficulty rising from a squatting position and mildly elevated serum creatine kinase levels (39). These individuals were initially referred to neurological clinics for assessment, prior to the diagnosis of a skeletal dysplasia following radiographic evaluation. The analysis of muscle biopsies often proved inconclusive (40). An in-depth analysis of the *Comp*^T585M^ model of mild PSACH/MED (39,41) revealed that the mild myopathy observed in patients, and recapitulated in this mouse model, was actually the result of an underlying tendinopathy that was manifesting primarily at the myotendinous and perimyseal junctions; this finding further correlated with joint laxity, which is another prominent clinical feature of PSACH and MED patients. Subsequent phenotypic analysis of the other two PSACH/MED mouse models (D469del COMP and V194D matrilin-3) revealed that both the myopathy and underlying tendinopathy were associated with mutant COMP, and not mutant matrilin-3, indicating that the neuromuscular complications in PSACH and MED are not just a side effect of the short limbed dwarfism alone (42). This in-depth phenotypic analysis of relevant genetic models of the PSACH-MED disease spectrum has yielded important information related to the specific site of the musculoskeletal complications and their underlying cause; both of which will be value aids for the diagnosis and management of these conditions.

## 11. Strengths and weaknesses of different genetic approaches to model skeletal diseases

The various transgenic approaches described in this review have both strengths and weaknesses as with all model systems. Homologous recombination allows the correct spatial and temporal expression of the mutant allele at levels comparable to the endogenous allele; however, mice are often required to be homozygous for the mutant allele to achieve a recognisable and quantifiable phenotype. This is in contrast to individuals with autosomal dominant PSACH and MED who only need to be heterozygous for the mutation. Posey *et al* (43) proposed this as weakness of the homologous recombination approach and suggested that inducible transgenic overexpression is required to produce a phenotype in mice that are heterozygous for the mutant allele; however, the random integration of multiple copies of the mutant transgene into the mouse genome is also not representative of the human condition. Furthermore, the inducible expression of mutant COMP also has its limitations since it is impossible to regulate the amount of dox ingested by individual transgenic mice and determining the levels of mutant COMP expression in the cartilage of each mouse is not feasible. Nevertheless, despite the differences of the two transgenic approaches, both mutant mouse lines demonstrated hallmark features of PSACH, which included mutant COMP retention, increased and dysregulated apoptosis with corresponding areas of hypocellularity (15,22,44). Moreover, cell proliferation was markedly reduced in both mouse models, which has recently been identified as a quintessential feature of growth plate dysplasia and reduced bone growth (11).

The use of endogenous *Matn3* and *Comp* promoters by the homologus recombination approach (7,15,19) has also allowed the in-depth analysis of a range of other musculoskeletal tissues that is not possible with *Col2a1*-driven transgenic overexpression. These mouse phenotyping studies have confirmed the importance of tendinopathy and mild myopathy to the overall pathophysiology of PSACH and MED that will ultimately provide greater understanding of the clinical presentation of these GSDs.

The development and deep phenotyping of ‘ER-stress phenocopies’ (30,31) is a novel genotype-independent approach for determining the role of ER stress in GSDs. The use of the *Col2a1* promoter generated a model that exhibited ER stress in a broad range of cartilaginous tissues including the growth plate and articular cartilages. The resulting phenotype, whilst not phenocopying any specific disease, provided a ‘generalised chondrodysplasia’ in which to identify fundamental disease mechanisms. In contrast, the use of the *Col10a1* promoter, which specifically targeted expression to hypertrophic chondrocytes, accurately phenocopied metaphyseal chondrodysplasias type Schmid. In conclusion, this genetic approach demonstrated that ER stress can be accurately modelled in either a single cell type (or differentiation state) or in an entire tissue and will provide a powerful technique for determining the role of ER stress in a broad group of inherited connective tissues diseases.

In conclusion, these different genetic mouse models (transgenic overexpression expression, knock-in by homologous recombination and novel phenocopies) of the PSACH-MED phenotypic spectrum have recapitulated key aspects of disease pathology and identified new fundamental mechanisms that have both temporal and spatially context within the cartilage growth plate. The acquisition of this new knowledge would not have been possible through the use of cell culture models, which do not recapitulate the dynamic properties of the cartilage growth plate.

## Figures and Tables

**Table I:** Human genetic skeletal diseases result from qualitative (anti-morphic) defects in cartilage structural proteins.

Gene	Protein	Disease(s)	Genetic loci	Domain	Refs.
*COMP*	COMP	Pseudoachondroplasia (AD) Multiple epiphyseal dysplasia (AD)	PSACH EDM1	T3-7 repeats C-terminal domain	(4,45)
*MATN3*	Matrilin-3	Multiple epiphyseal dysplasia (AD)	EDM5	vWFA	(18)
*COL9A1* *COL9A2* *COL9A3*	Type IX collagen	Multiple epiphyseal dysplasia (AD)	EDM6 EDM2 EDM3	COL3 domain	(46–48)
*COL2A1*	Type II collagen	Diverse range of AD and AR phenotypes collectively known as type II collagenopathies	Various	Triple helical region and C-propeptide	(49)
*COL11A1* *COL11A2*	Type XI collagen	Diverse range of AD and AR phenotypes collectively known as type XI collagenopathies	Various		(50)
*COL10A1*	Type X collagen	Metaphyseal chondrodysplasia, type Schmid (AD)	MCDS	Carboxyl-terminal non-collagenous domain (NC1)	(51)
*ACAN*	Aggrecan	Spondyloepimetaphyseal dyslasia (AR) Osteochondritis dissecans (AD) Short stature, accelerated bonematuration (AD)	SEMD OCD	G3 C-type lectin domain	(52–54)

COMP, cartilage oligomeric matrix protein; PSACH, pseudoachondroplasia; AD, autosomal dominant; AR, autosomal recessive.

**Table II:** Mouse models of the PSACH-MED disease spectrum and novel phenocopies to model ER stress in the cartilage growth plate.

Disease	Gene	Mutation	Approach taken	Promoter	Refs.
PSACH	*COMP*	D469del	Transgenic (rat COMP cDNA)	*Col2a1*	(20)
PSACH	*COMP*	D469del	Transgenic (human COMP gene)	Native	(3)
PSACH	*COMP*	D469del	Transgenic (human COMP cDNA)	*Col2a1*	(3)
PSACH	*COMP*	D469del	Transgenic inducible overexpression (human COMP cDNA)	*Col2a1* and tetracycline responsive element	(21,22,44)
PSACH	*COMP*	D469del	Knock-in	Native	(9,15,42)
PSACH-MED	*COMP*	T585M	Knock-in	Native	(7,9,38,41)
MED	*MATN3*	V194D	Knock-in	Native	(9,19,23,42)
Chondrodysplasia	*TG*	*Rdw* (G2320R)	Transgenic phenocopy	*Col2a1*	(31)
Chondrodysplasia	*TG*	*Cog* (L2293P)	Transgenic phenocopy	*Col2a1*	(55)

ER, endoplasmic reticulum; COMP, cartilage oligomeric matrix protein; PSACH, pseudoachondroplasia; MED, multiple epiphyseal dysplasia.

**Table III:** Key pathological and quantitative measures of disease severity in various knock-in mouse models of PSACH-MED and novel ER stress phenocopies.

Model	Age at onset	Final reduction in bone length			Cell proliferation	Apoptosis	Protein retention	ER stress	Stress pathway
		3 weeks	6 weeks	9 weeks	3 weeks	3 weeks			
*Comp* ^D469del^	~6 weeks	No differences	↓ 6%	↓ 5%	↓ 17%	↑ 90-fold (Pz) ↑ 5-fold (Hz)	Yes	Yes	Novel (APR/EOR)
*Comp* ^T585M^	~9 weeks	No differences	No differences	↓ 5%	↓ 24%	↑ 3-fold (Rz) ↑ 12-fold (Pz) ↑ 3-fold (Hz)	Slight	Mild	Mild UPR
*Matn3* ^V194D^	~2 weeks	↓ 12%	↓ 13%	↓ 12%	↑ 16%	Spatially dysregulated	Yes	Yes	UPR
*Col2*-Tg^Rdw^	At birth	↓ 8%	↓ 5%	↓ 5%	↑ 21%	No differences	Yes	Yes	UPR
*Col2*-Tg^cog^	~3 weeks	↓ 7%	↓ 4%	↓ 4%	↑ 12%	No differences	Yes	Yes	Novel

EOR, ER overload response; ER, endoplasmic reticulum; APR, aggregated protein response; UPR, unfolded protein response; Pz, proliferative zone; Hz, hypertrophic zone; Rz, resting zone; PSACH, pseudoachondroplasia; MED, multiple epiphyseal dysplasia.

## Abbreviations

COMP: cartilage oligomeric matrix protein
GSD: genetic skeletal disease
PSACH: pseudoachondroplasia
MED: multiple epiphyseal dysplasia
ER: endoplasmic reticulum
UPR: unfolded protein response
EOR: ER overload response
APR: aggregated protein response
ECM: extracellular matrix
CTD: carboxyl-terminal domain
vWFA: von Willebrand Factor A
Xbp1: X-box binding protein 1
BiP: binding immunoglobulin protein
CHOP: C/EBP-homologous protein
FACIT: fibril-associated collagens with interrupted triple helices
